# Relationship between leadership-member exchange (LMX) and flow at work among medical workers during the COVID-19: the mediating role of job crafting

**DOI:** 10.1186/s40359-023-01194-3

**Published:** 2023-05-18

**Authors:** Huazhen Ji, Xiaoyun Zhao, Junhua Dang

**Affiliations:** 1 Xuzhou Medical University, Xuzhou, China; 2 Faculty of Education, Huaibei Normal University, Huaibei, China; 3 Anhui Engineering Research Center for Intelligent Computing and Application on Cognitive Behavior (ICACB), Huaibei, Anhui China; 4 Institute of Social Psychology, School of Humanities and Social Sciences, Xi’an Jiaotong University, Xi’an, China; 5 Uppsala University, Uppsala, Sweden

**Keywords:** Medical workers, Leader-member exchange, Job crafting, Flow at work, Gender

## Abstract

Based on relational leadership theory and self-determination theory, this study aims to investigate the relationship between leader-member exchange (LMX), job crafting, and flow at work among medical workers in the context of the COVID-19 pandemic. Participants in the study consisted of 424 hospital employees. The results showed that: (1) the LMX positively predicted flow at work; (2) two types of job crafting (increasing structural job resources and challenging job demands) played a mediating role between the LMX and flow at work; and (3) gender did not moderate these mediating effects as suggested by previous studies. These results indicate that the LMX can not only directly predict flow at work, but also indirectly predict work-related flow through job crafting by increasing structural job resources and challenging job demands, thus providing new insights for enhancing flow experiences of medical workers.

## Introduction

Medical workers play a critical role in traditional treatment or prevention of diseases, medical personnel training, and medical science and technology development. In the face of major infectious disease threats and the fight against major natural disasters such as the COVID-19, medical workers always actively participate in rescue work at the risk of their lives. Notably, the coronavirus pandemic has dramatically changed medical staffs’ working process and increased their workload and work stress, which negatively impacted their working experience and mental health [1, 2]. In view of this, it has become an important topic to create a good working atmosphere for medical workers and promote their positive working experience.

With the development of positive psychology, the importance of a new working experience, namely flow at work, has been recognized. It refers to a short period of peak experience that occurs during the working process, including concentration, enjoyment, and intrinsic working motivation [3]. Researchers have shown that employees with high levels of flow experiences had better working performance [3] as well as more psychological capital and creativity [4]. Moreover, Sweeny et al. reported that employees with flow experiences were more able to alleviate the low happiness and depression caused by the COVID-19 pandemic [5].

Given the significant role of flow at work as a positive experience in organizational behavior studies, it is important to investigate how it can be improved. According to relational leadership theory, the quality of the relationship between leaders and employees is crucial for an employees’ working experience and leader-employee interaction has a greater impact on working achievements than many other factors such as employees’ characteristics [6]. One of the key concepts in this interaction is the leader-member exchange (LMX). Existing studies show that the LMX is strongly related to positive work experiences such as employees’ job satisfaction and working engagement [7, 8]. However, the predictive effect of the LMX and flow at work has not been investigated in the literature, and the mechanisms underlying this relationship are also unknown. Therefore, the current research aims to examine whether the LMX is a significant predictor of flow experiences at work. Meanwhile, we also aim to explore the psychological mechanisms underlying this relationship and the boundary conditions of this relationship. Specifically, we will test whether job crafting can mediate the relationship between the LMX and flow at work and whether gender can moderate this relationship. The rationales are presented below.

## Theoretical background and hypothesis

### The predictive effect of LMX on flow at work

The LMX theory differs from other leadership theories in that it emphasizes the dyadic relationship between the leader and the subordinate. Leaders do not interact with all subordinates equally. They develop and maintain different types of exchange relationships with employees of the same workgroup. In this respect, LMX relationships exist on a continuum, ranging from low quality to high quality [9]. A high quality LMX can result in employees feeling a sense of trust toward the organization and willing to participate in various organizational activities [10]. In contrast, employees with a low quality LMX receive limited support, low levels of trust, and relatively few resources from leaders, therefore they are more likely to develop negative working attitudes [11]. According to previous studies, compared with employees with a low quality LMX, those with a high quality LMX have a greater degree of engagement and dedication [12].

Similarly, the LMX is also likely to be positively associated with flow experiences. Flow at work mainly includes concentration, enjoyment, and intrinsic working motivation [3]. Concentration refers to a person’s capacity to focus so intently on the task that he/she loses track of what is going on around him/her. Enjoyment indicates that the work itself brings positive emotion and cognitive evaluations, allowing the individual to enjoy the quality and pleasure of the work. Intrinsic work motivation is the individual’s will to devote himself to the work itself with a strong sense of working value and satisfaction, rather than being influenced by external stimulation (e.g., rewards and promotions).

Self-determination theory suggests that individuals have an innate tendency to internalize external motivation; and that the external environment can provide nurturing conditions for individuals to internalize their external reason and thus generate autonomous motivation, which in turn will further promote proactive behavior [13]. According to this theory, employees with a high quality LMX are given more freedom and flexibility at work. Their autonomy needs are greatly satisfied, which further induces their autonomous behaviors, which are important source of flow at work [14]. Therefore, a high quality LMX is likely to generate more flow experiences, mainly through employees’ autonomous behaviors. One typical example of this autonomous behavior is job crafting, a self-need-oriented proactive behavior [15]. We elaborate this mechanism below.

### The mediating role of job crafting

#### Job crafting

Differing from the traditional top-down job redesign approach in which leaders create jobs and roles, the bottom-up, employee-initiated job redesign approach such as job crafting has emerged as a new perspective in job design theory. It is seen as a specific form of proactive working behavior that employees engage in to adjust their job to their needs, skills, and preferences [16]. Based on the Job Demand-Resources Model (JD-R Model), Tims et al. classified job crafting into four different types of behaviors: (1) increasing structural job resources (e.g., gaining more development opportunities and autonomy), (2) increasing social job resources (e.g., gaining support and feedback from leaders), (3) increasing challenging job demands (e.g., increasing task content, volunteering for projects of interest), and (4) decreasing hindering job demands (e.g., reducing work content, prioritizing important tasks) [17].

#### LMX predicts job crafting

According to the JD-R Model, job resources within an organization facilitate the generation of crafting behaviors [18]. Only when the organization has relevant supportive resources and conditions, can employees have sufficient space and motivation to voluntarily craft their jobs. A high quality LMX is a powerful emotional resource in an organization. Employees with a high quality LMX can perceive stronger leadership support, show greater self-express willingness, and have more positive work adjustment ideas [19]. Therefore, a high quality LMX tends to stimulate more crafting behaviors.

#### Job crafting predicts flow at work

When crafting their jobs, employees may actively choose tasks and negotiate different job contents. During this process, they can create opportunities to pursue passions or engage in activities and topics that spark deep interest, which is a rich source of concentration, enjoyment, and intrinsic motivation (the key components of flow at work). For example, Tims et al. found that job crafting was positively related to employees’ absorption, vigor, and dedication rated by other colleagues [17]. Harju et al. found that seeking challenges at work, one type of job crafting, could prevent boredom, an unpleasant state characterized by attention difficulties [20]. Daily diary studies also revealed that employees reported more flow experience at work and enjoy their work more on days they engaged in job crafting behaviors [21]. Therefore, job crafting can predict flow experience at work.

#### LMX predicts flow at work via job crafting

As we showed before, a high quality LMX is a powerful emotional resource in an organization, which can stimulate more crafting behaviors. When crafting their jobs, employees can engage in activities and topics that spark deep interest, through which they immerse themselves in the work. More importantly, self-determination theory holds that people have three basic psychological needs: competence, relatedness, and autonomy. The satisfaction of basic psychological needs can promote the best physical, psychological, and social functions [13]. According to the self-determination view of flow at work, employees experience flow when their basic needs are satisfied [14]. Therefore, satisfaction of basic needs serves as an antecedent of flow at work, which was supported by many longitudinal studies that had causal implications. For example, Fullagar and Kelloway examined the relationships between five types of job characteristics (skill variety, task identity, task significance, autonomy, and feedback) and flow at work in a longitudinal study [22]. They found job autonomy could uniquely and positively predict subsequent flow experiences. Meanwhile, a two-wave longitudinal study of schoolteachers showed that initial self-efficacy (satisfaction of competence) positively predicted following flow experiences [23]. Job crafting is such an effective strategy that can greatly satisfy basic needs. By seeking challenges to promote self-growth, adjusting the job content in line with passions or values, and influencing whom they interact with while doing the job, job crafting plays to satisfaction of competence, autonomy, and relatedness, respectively [14]. Therefore, the LMX is an important job resource that gives employees more freedom and flexibility. Supported by a high quality LMX, employees have more chances to and are more willing to engage in job crafting. Through job crafting, their basic psychological needs are satisfied, which in turn brings them flow experiences. Therefore, we suggest that job crafting may mediate the relationship between the LMX and flow at work.

### The moderating role of gender

Gender difference has been a hot topic in the field of organizational behavior. Current research has shown that the effect of the LMX on employee work behaviors varied by gender. For example, Wang et al. found that females in a high quality LMX exhibited more positive work behaviors than males [24]. Kim Jin and Kim Jong demonstrated that the LMX were more strongly related to life satisfaction in men than women [25]. Meanwhile, job crafting may also vary by gender. A meta-analysis on job crafting found that female employees showed more crafting behaviors than male employees, and females were more likely than males to craft via increasing structural job resources and increasing challenging job demands [26]. In the light of the gender difference described above, this study will also test whether gender moderate the relationship between the LMX and flow experiences and the mediating effect of job crafting.

## Research methods

### Participants

We focused on medical workers (i.e., doctors and nurses) who were formally affiliated with public hospitals. The data were collected through online and offline questionnaires between June and July 2020. The first author worked in a medical university and had social connections that helped questionnaire delivery. Regarding the offline delivery, we reached 300 medical workers from three public hospitals in Anhui Province, China. They fulfilled the paper-pencil questionnaire. Regarding the online delivery, we reached 124 medical workers who were distributed in two provinces of China (Shandong and Anhui). They were contacted by the first author and filled the same questionnaire online via a link sent to them. In total, there were 424 valid respondents, including 187 (44.1%) males and 237 (55.9%) females, and 146 (34.4%) doctors and 278 (65.6%) nurses. The age of the subjects ranged from 20 to 55 years (*M* = 34.65 years, *SD* = 7.48).

### Measures

#### Leader-member exchange scale

A unidimensional scale of leader-member exchange (LMX) developed by Liden and Graen [27] was used. It includes 7 items and is scored on a 5-point Likert scale, with higher scores indicating a better leadership-member exchange relationship. The Cronbach’s alpha coefficient of the scale in this study was 0.86.

#### Job crafting scale

The job crafting scale, developed by Tims et al. [17], was used. The scale consists of 21 items with 4 dimensions (increasing structural work resources, increasing social work resources, increasing challenging work demands, and decreasing hindering work demands). It is scored on a 5-point Likert scale from “never” to “always”. The higher the score, the more positive the individual’s work reinvention behavior. In this study, the Cronbach’s alpha coefficient of the scale was 0.92.

#### Flow at work scale

Bakker et al. designed a flow at work scale [28]. The measure is rated on a 7-point Likert scale, with the higher scores indicating greater flow experiences at work. In this investigation, the scale’s Cronbach’s alpha coefficient was 0.94.

### Data analysis

The raw data were effectively organized and entered. Descriptive statistical analysis and correlation analysis were performed by SPSS software, version 25.0. The mediation and moderation analysis were performed by the PROCESS plug-in.

## Results

### Main effect of LMX

As can be seen in Table 1, the LMX and each dimension of job crafting have a significant positive correlation with flow at work (*p* < 0.01); there was also a significant positive correlation between the LMX and each dimension of job crafting (*p* < 0.01). We conducted multiple regression to test the main effect of the LMX on flow at work by controlling for the demographic variables. As shown in Table 2, in Step 1, we entered the demographic variables, which explained 7% of the total variance. In Step 2, we entered the LMX, which explained additional 27% variance beyond the demographic variables, suggesting that the LMX is an important predictor of flow at work.

**Table 1:** Descriptive statistics and correlation analysis for each variable

	M	SD	1	2	3	4	5	6	7	8	9
Age	34.65	7.48	1								
Gender	0.44	0.50	-0.12^*^	1							
Position	0.66	0.48	0.01	-0.61^**^	1						
LMX	3.54	0.71	0.06	-0.03	-0.09	1					
Increasing structural JR	4.00	0.64	0.12^*^	-0.06	-0.10	0.45^**^	1				
Increasing challenging JD	3.41	0.79	0.06	-0.03	-0.11^*^	0.58^**^	0.74^**^	1			
Increasing social JR	3.46	0.67	-0.05	-0.03	-0.10^*^	0.54^**^	0.58^**^	0.68^**^	1		
Decreasing hindering JD	3.55	0.63	0.05	-0.06	-0.10^*^	0.40^**^	0.66^**^	0.61^**^	0.60^**^	1	
Flow at work	4.89	1.30	0.12^*^	-0.03	-0.18^**^	0.55^**^	0.57^**^	0.66^**^	0.50^**^	0.42^**^	1

Note: Gender: 0 = female, 1 = male; Position: 1 = doctor, 2 = nurse; JR: job resources; JD: job demands. *p < 0.05, **p < 0.01, ***p < 0.001

**Table 2:** Multiple regression of flow at work on demographic variables and the LMX.

	Flow at work		
	*β*	*t*	Δ*R*^*2*^
Step 1			0.07
Age	0.10	2.07*	
Gender	− 0.20	-3.34***	
Position	− 0.30	-5.03***	
Step 2			0.27
LMX	0.52	12.983***	

Note: *p < 0.05, **p < 0.01, ***p < 0.001

### Mediating effects with moderation

To verify the mediating effect of job crafting in the relationship between the LMX and flow at work, as well as the moderating effect of gender in all the relationships verified by the mediating model, we conducted the mediation analysis and the moderated mediation analysis separately using the PROCESS macro, version 3.2, for SPSS. Of these, the number of bootstrap samples for bias-corrected bootstrap confidence intervals is 5000, and the level of confidence for all confidence intervals in output is 95%. All study variables were standardized before analysis.

**Fig. 1:**
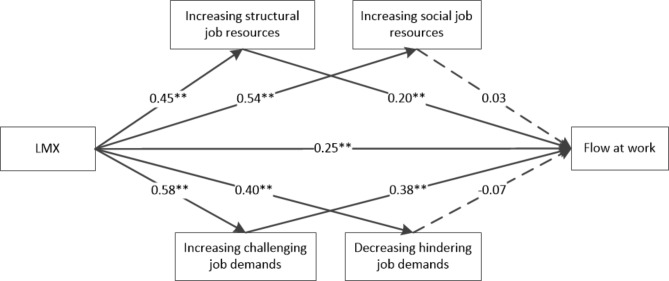
The mediating role of job crafting between the LMX and flow at work

First, the mediation analysis was performed using Model 4 in PROCESS. The results found (see Fig. 1) that both the total effect and the direct effect of LMX on flow at work were significant (total effect = 0.55, 95%CI = [0.47, 0.63], *p* < 0.001; direct effect = 0.25, 95%CI = [0.16, 0.33], *p* < 0.001). The indirect effects of increasing structural job resources (effect = 0.09, 95%CI = [0.04, 0.15]) and increasing challenging job demands (effect = 0.22, 95%CI = [0.15, 0.31]) on the relationship between the LMX and flow at work were significant, accounting for 16.70% and 40.33% of the total effect, respectively. However, the indirect effects of increasing social job resources (effect = 0.03, 95%CI = [-0.08, 0.15]) and decreasing hindering job demands (effect = -0.05, 95%CI = [-0.14, 0.03]) were not significant, with 95% confidence intervals containing “0”.

Second, the moderated mediation analysis was conducted using Model 59 in PROCESS, which incorporated the gender variable (0 = female and 1 = male) into the model. It was found that none of the interaction terms between gender and the predictors was significant (*p* > 0.05), indicating that the moderating effects of gender did not hold (see Table 3).

**Table 3:** Testing the moderated mediation

	Flow at work		Increasing structural JR		Increasing challenging JD		Increasing social JR		Decreasing hindering JD		Flow at work	
	*β*	*t*	*β*	*t*	*β*	*t*	*β*	*t*	*β*	*t*	*β*	*t*
LMX	0.55	13.50^**^	0.40	6.86^**^	0.53	9.87^**^	0.48	8.68^**^	0.36	5.98^**^	0.27	4.77^**^
Gender			-0.09	-1.01	-0.03	-0.38	-0.02	-0.23	-0.10	-1.13	-0.01	-0.07
LMX×Gender			0.11	1.22	0.09	1.18	0.13	1.64	0.08	0.89	-0.06	-0.69
Structural JR											0.27	3.55^**^
Challenging JD											0.30	3.77^**^
Social JR											0.08	1.22
Hindering JD											-0.14	-2.15^*^
Structural JR×Gender											-0.14	-1.21
Challenging JD×Gender											0.18	1.44
Social JR×Gender											-0.10	-0.93
Hindering JD×Gender											0.17	1.62
*R* ^*2*^	0.3		0.21		0.34		0.30		0.16		0.50	
*F*	182.26^***^		37.48^***^		70.76^***^		60.07^***^		27.65^***^		0.16^***^	

Note: *p < 0.05, **p < 0.01, ***p < 0.001

## Discussion

### Theoretical implications

First, based on relational leadership theory, this study demonstrated the positive effect of the LMX on flow at work, which not only confirms the point that relational elements of organizational structure influence employees’ work perceptions [29], but also extends the boundaries of relational leadership theory. Compared with other work experiences related to relational leadership theory, flow at work is more emphasized as a short-term peak experience, which is thought to be a significant contributor to not only feelings of well-being and happiness but also work performance and creativity [3, 4]. During the COVID-19 pandemic, flow experiences of medical staffs are of particular significance for relieving their mental stresses [5]. Therefore, it is crucial to investigate the antecedents of flow at work, which would have important practical implications for the organizations.

Second, this study found that increasing structural job resources mediated the relationship between the LMX and flow at work. Although the LMX positively predicted increasing social resources, the latter had no mediating effect between the LMX and flow at work. This result coincides with the viewpoint of self-determination theory. That is, internal motivation is related to job engagement while external motivation is not. Structural job resources reflect the employees’ motivation to develop professionally and originate from “within” the employees [12]. Those with a high quality LMX tend to have a better working environment, and to pursue more career development opportunities and work autonomy (structural job resources), which strengthens their determination to realize themselves, makes them form positive expectations for work, and continuously immerses themselves in work. On the contrary, social job resources reflect employees’ external motivations for career development. Although a high quality LMX may enable employees to have more opportunities to get leadership support and feedback (social job resources), too much pursuit of the realization of external resources is not beneficial for employees to comprehend the value of the work itself, thus having little contribution to flow experiences in the work [30].

In addition, this study also revealed increasing challenging job demands mediated the relationship between the LMX and flow at work. Although the LMX positively predicted decreasing hindering job demands, the latter had no mediating effect between the LMX and flow at work. This result is partially consistent with the two-dimensional work stressor framework [31], which puts forward two kinds of job demands: challenging job demands and hindering job demands. Challenging job demand is positively correlated with the result of motivation, while hindering job demand is not. A high quality LMX creates a challenging working environment for employees, enhances the attractiveness of the work, and develops their knowledge and skills [32]. This helps to motivate employees to work, so that they can be more engaged and actively respond to and solve problems. On the contrary, decreasing hindering job demands is an avoidance-oriented coping mechanism. This self-protection strategy is related to avoiding making mistakes rather than actively engaging in a task, thus contributing little to flow experiences in the work [33].

Third, this study indicated that gender did not moderate the mediating effect of job crating between the LMX and flow at work. The results described above refute the traditional view of gender roles that females are at a disadvantage in the workplace compared with males. In fact, as the social roles of females change, more and more females are involved in work, and many of them have achieved management and leadership positions [34]. Therefore, as the traditional view of gender roles has changed, and the status of males and females in the workplace tends to be equal.

### Practical insights

The practical implications of this study are as follows. On the one hand, hospital leaders should treat every subordinate fairly and advocate a fair and open management atmosphere. Leaders are encouraged to create a perception of insider status for their subordinates and give more emotional encouragement and care, so their subordinates feel respected, trusted, and motivated to put more into work. On the other hand, hospital leaders should give their staffs a certain degree of autonomy in their work, offer more opportunities for further education and training outside the hospital, and appropriately set tasks with a certain degree of challenge to enhance their interest in their work. This is particularly important in stressful situations, such as the current COVID-19 pandemic. In these situations, employees are more likely to be distracted by worries, anxieties, and many other work-unrelated factors. Autonomous job crafting is not only beneficial for promoting work engagement and performance but also alleviating the negative impact of unpleasant feelings and distractions [20]. It is also important that hospital managers abandon the inherent gender concept and adopt appropriate management strategies according to the gender and other characteristics of staffs, so as to stimulate high-quality job crafting among medical workers.

### Limitations and future studies

On the one hand, although this study administered tests to employees in the healthcare system, participants’ positions were not differentiated. To reduce homogenous errors, paired data information on leaders and subordinates could be included in the future. On the other hand, this study took a cross-sectional approach. Although the results reflect to some extent a causal relationship between the tested variables, it needs to be confirmed by longitudinal research and experimental research in the future. In addition, because it was not easy to recruit medical workers during the pandemic, our sample size was relatively small. Future studies may consider larger sample sizes that can provide stronger statistical power.

## Conclusion

This study confirmed the positive predictive effect of the LMX on flow at work and the mediating effects of two types of job crafting, increasing structural job resources and increasing challenging job demands. This suggests that medical workers with a high quality LMX are more likely to generate flow experiences by increasing structural job resources and increasing challenging job demands. However, this process is unaffected by gender. In conclusion, these findings provide new insights for enhancing flow experiences of medical workers.

## Data Availability

The datasets used during the current study available from the corresponding author on reasonable request.
